# Post-magmatic fracturing, fluid flow, and vein mineralization in supra-subduction zones: a comparative study on vein calcites from the Troodos ophiolite and the Izu–Bonin forearc and rear arc

**DOI:** 10.1007/s00531-020-01978-7

**Published:** 2021-02-13

**Authors:** Dennis Quandt, W. Kurz, P. Micheuz

**Affiliations:** 1 Institute of Earth Sciences, NAWI Graz Geocenter, University of Graz, Graz, Austria; 2 Present Address: Division of Structural Geology and Tectonics, Institute of Applied Geosciences, Karlsruhe Institute of Technology, Karlsruhe, Germany

**Keywords:** Troodos ophiolite, Izu–Bonin forearc, Calcite veins, Microtextures, Geochemistry

## Abstract

Based on the published data of pillow lava-hosted mineralized veins, this study compares post-magmatic fracturing, fluid flow, and secondary mineralization processes in the Troodos and Izu–Bonin supra-subduction zone (SSZ) and discusses the crucial factors for the development of distinct vein types. Thin section and cathodoluminescence petrography, Raman spectroscopy, fluid inclusion microthermometry, and trace element and isotope (^87^Sr/^86^Sr, δ^18^O, δ^13^C, Δ_47_) geochemistry indicate that most veins consist of calcite that precipitated from pristine to slightly modified seawater at temperatures < 50 °C. In response to the mode of fracturing, fluid supply, and mineral growth dynamics, calcites developed distinct blocky (precipitation into fluid-filled fractures), syntaxial (crack and sealing), and antitaxial (diffusion-fed displacive growth) vein microtextures with vein type-specific geochemical signatures. Blocky veins predominate in all study areas, whereas syntaxial veins represent subordinate structures. Antitaxial veins occur in all study areas but are particularly abundant in the Izu–Bonin rear arc where the local geological setting was conducive of antitaxial veining. The temporal framework of major calcite veining coincides with the onset of extensional faulting in the respective areas and points to a tectonic control on veining. Thus, major calcite veining in the Troodos SSZ began contemporaneously with volcanic activity and extensional faulting and completed within ~ 10–20 Myr. This enabled deep seawater downflow and hydrothermal fluid upflow. In the Izu–Bonin forearc, reliable ages of vein calcites point to vein formation > 15 Myr after subduction initiation. Therefore, high-T mineralization (calcite, quartz, analcime) up to 230 °C is restricted to the Troodos SSZ.

## Introduction

Rocks and geological structures exposed in ophiolites and drill cores recovered during the International Ocean Discovery Program (IODP) and its predecessors provide insights into the tectono-magmatic evolution and architecture of the oceanic crust (Dilek et al. 2000). Subsequent to its formation, the physical and chemical properties of the oceanic crust are modified by different, interacting processes such as faulting and fracturing, fluid circulation, fluid–rock interaction, secondary mineralization, and seafloor sedimentation (e.g., Anderson and Hobart 1976; Thompson 1983; Alt 1995; Fisher 1998; Fisher and Becker 2000; Bach et al. 2004; Spinelli et al. 2004; Wilcock and Fisher 2004; Coogan and Gillis 2018). Extensional faults and fractures substantially increase the permeability of the oceanic crust, channelize advective fluid flow into depth, and focus fluid-mediated heat dissipation to the seafloor (e.g., Lowell 1975; Fisher 1998; Fisher and Becker 2000). Circulating fluids may interact with ambient rocks altering the geochemical compositions of rocks and fluids (e.g., Thompson 1983; Bach et al. 2004). Secondary minerals that precipitate from these fluids inherit the geochemical fluid signature (e.g., Bau and Möller 1992; Lottermoser 1992) and vein microtextures document the mode of fracturing and fluid flow (e.g., Bons et al. 2012). Secondary mineralization and a sedimentary cover, however, decrease the crustal permeability and act as a thermal and hydraulic seal for the oceanic basement (e.g., Anderson and Hobart 1976; Spinelli et al. 2004; Coogan and Gillis 2018). These post-magmatic processes are the subject of this paper in which we compare published data of mineralized veins from the Late Cretaceous Troodos ophiolite (Cyprus) and the Early Eocene Western Pacific Izu–Bonin forearc and rear arc (Alt et al. 1998; Gillis et al. 2015; Quandt et al. 2018, 2019, 2020a; Weinzierl et al. 2018; Coogan et al. 2019). This data set comprises (1) petrographic observations based on thin section and cathodoluminescence (CL) microscopy, (2) mineralogical determinations concluded from Raman spectroscopy, (3) mineral formation temperatures derived from δ^18^O, clumped isotope (Δ_47_), and fluid inclusion thermometry, (4) parental fluid compositions inferred from rare earth element and yttrium (REE + Y) and isotope (^87^Sr/^86^Sr, δ^18^O, δ^13^C, Δ_47_) geochemistry, (5) and relative ages of vein calcite precipitation deduced from ^87^Sr/^86^Sr stratigraphy. These data are complemented by new electron microprobe element mappings.

Based on the analysis of similarities and differences between mineralized veins from the different locations, we compare how fracturing, fluid flow, and secondary mineralization modified the respective crustal sections. Although both study areas are spatially and temporally unrelated, this represents a suitable approach for a comparative study since these crustal sections equally reveal complete, well-preserved, and extensively veined volcanic sequences that formed in similar supra-subduction zone settings (SSZ; Gass 1968; Moores and Vine 1971; Pearce and Robinson 2010; Reagan et al. 2017, 2019; Woelki et al. 2018, 2019, 2020). In addition, the respective volcanic units are sufficiently old (~ 94–90 and ~ 52–47 Ma, respectively) to expect termination of major secondary mineralization (Blome and Irwin 1985; Mukasa and Ludden 1987; Ishizuka et al. 2018; Reagan et al. 2019; Chen et al. 2020; Morag et al. 2020), which is believed to be largely completed (> 80%) within ~ 25 Myr after crust formation (Coogan and Gillis 2018). We also discuss the crucial factors for the formation of different vein types and their geochemical signatures considering the modes of fracturing and fluid supply as well as the local geological environment. Hence, this comparative study provides a robust basis to test if specific vein types show distinct geochemical signatures and thus contributes to the understanding of vein formation within the volcanic units of the oceanic crust in general.

## Geological background

**Fig. 1:**
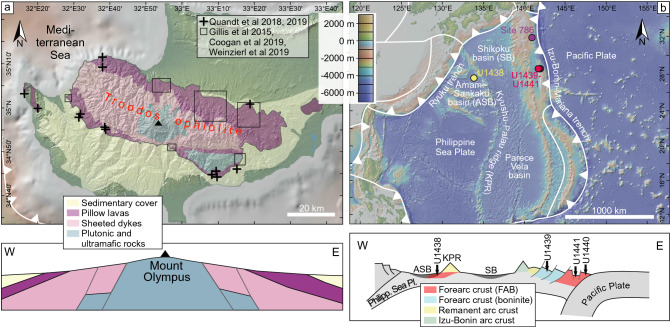
Simplified geological maps and cross sections (not to scale) of the **a** Troodos ophiolite and **b** Izu–Bonin forearc/rear arc. Maps and cross sections are based on Constantinou (1995), Kurz et al. (2019), and Ring and Pantazides (2019) and were created using GeoMapApp (Ryan et al. 2009)

The Neo-Tethyan Troodos ophiolite and drill cores from the Western Pacific Izu–Bonin forearc and rear arc recovered during IODP Expeditions 352 and 351 (Arculus et al. 2015a; Reagan et al. 2015) reveal volcanic rock successions that formed in Late Cretaceous and Early Eocene SSZ forearc settings, respectively (Fig. 1; Pearce and Robinson 2010; Reagan et al. 2017, 2019; Woelki et al. 2018, 2019, 2020). Both tectonic settings are similarly characterized by an extensional regime above a subducting slab (Pearce and Robinson 2010; Reagan et al. 2019; Woelki et al. 2018, 2019, 2020). The geological development of both SSZ is described hereinafter and summarized in Table 1.

**Table 1:** Summary of the Troodos and Izu–Bonin SSZ settings

	Troodos ophiolite	Izu–Bonin forearc	Izu–Bonin rear arc
Location	Neo-Tethys	Western Pacific	Western Pacific
Tectonic setting	Spreading ridge above subduction zone	Subduction initiation	Subduction initiation
Duration of major volcanic activity	~ 94–90 Ma	~ 52–50 Ma	~ 49–47 Ma
Volcanic stratigraphy	Tholeiitic basalts–dacites, boninites	FAB and boninites	FAB-like rocks
Melting	Fluid-induced, possibly decompression	Decompression, then fluid-induced	Decompression
Estimated spreading rate	Ambiguous, alternating slow and intermediate/fast rates (?)	Poorly constrained, intermediate–fast (?)	Poorly constrained, intermediate–fast (?)
Onset of sedimentation	~ 91 Ma	~ 35 Ma	~ 49 Ma
Sedimentary succession	Umbers, radiolarian mudstones, volcaniclastic mudstones and sandstones, pelagic carbonates	Tuffaceous sediments, mudstones, siltstones, claystones, carbonates	Tuffaceous mudstones, claystones, sandstones
Major fault systems	Normal faults, grabens, detachments	Normal faults, grabens	Not observed
Time of faulting	~ 94-? Ma, during or shortly after cessation of spreading	< 35 Ma	No major faults observed
Slab rollback	~ 95–90 Ma	~ 35–15 Ma	None
Uplift	28–14 and 2.5–0.9 Ma	None	None

See text for references

### Troodos ophiolite

In previous studies, the formation of the oceanic crust now exposed in the Troodos massif was attributed to various tectonic models that were progressively modified over time (e.g., Gass 1968; Miyashiro 1973; Pearce 1975; Schmincke et al. 1983; Pearce et al. 1984; Rautenschlein et al. 1985; Flower and Levine 1987; Pearce and Robinson 2010; Regelous et al. 2014; Woelki et al. 2018, 2019, 2020). Current studies agree on the formation in an extensional SSZ forearc setting at a spreading axis within the southern Neo-Tethys between Africa and Eurasia (e.g., Pearce and Robinson 2010; Woelki et al. 2018, 2019, 2020). This setting is characterized by a spreading ridge located above a subducting slab that generated a complete and well-preserved Penrose-type stratigraphy. From bottom to top the ophiolitic stratigraphy consists of serpentinites, gabbros and local plagiogranites, sheeted dykes, pillow lavas, and a sedimentary cover (Gass 1968; Moores and Vine 1971; Anonymous 1972).

Radiolarian biostratigraphy of localized sedimentary rocks (~ 91–89 Ma) intercalated with and overlying the pillow lavas (Blome and Irwin 1985) as well as U–Pb geochronology of zircons (~ 94–90 Ma) from plagiogranites (Mukasa and Ludden 1987; Chen et al. 2020; Morag et al. 2020) constrained the formation of the Troodos SSZ crust to late Cenomanian/Turonian times.

Based on their stratigraphic positions as well as geochemical compositions, some authors subdivided the pillow lavas into a lower basic–acid and an upper ultrabasic–basic series (Gass 1968; Moores and Vine 1971) associated with spreading and subsequent subduction, respectively (Dilek et al. 1990; Thy and Esbensen 1993). Other authors interpreted the pillow lava units as on-axis and slightly off-axis eruptions from the same spreading center (Gass and Smewing 1973; Boyle and Robertson 1984). A recent study subdivided the pillow lavas into boninites as well as tholeiitic basalts, andesites, and dacites and confirmed the presence of two volcanic rock series (Woelki et al. 2018). However, the interbedding of both series, each enriched in slab-derived fluid-mobile elements, and the lack of any systematic compositional change with stratigraphic depth contradicted earlier studies (Woelki et al. 2018, 2019, 2020).

The Troodos ophiolite exposes several extensional structures such as extensional joints between sheeted dykes, listric normal faults, low-angle detachments, and grabens associated with spreading processes (Hurst et al. 1994; Varga et al. 1999). Hydrothermal mineralization along extensional faults indicates that faulting and hydrothermal circulation were active during spreading or shortly after its cessation (Dilek and Eddy 1992; Hurst et al. 1994; Varga et al. 1999) and probably occurred on-axis as well as off-axis (Schiffman et al. 1987; Schiffman and Smith 1988; Eddy et al. 1998; Prichard and Maliotis 1998). Some authors proposed a slab rollback model for the Troodos ophiolite in which they considered the extensional structures and magmatic record (Dilek and Flower 2003; Dilek and Furnes 2009; Pearce and Robinson 2010).

Based on different objects of investigation, indications for the spreading regime are ambiguous and estimates range from slow (Varga and Moores 1985; Dilek and Eddy 1992; Abelson et al. 2001) to intermediate/fast rates (Boyle and Robertson 1984; Allerton and Vine 1987, 1991). For instance, extensional structures indicate a slow spreading regime (e.g., Varga and Moores 1985), but the style of detachment faulting and crustal architecture differ from typical slow spreading mid-ocean ridges (e.g., Allerton and Vine 1987). This apparent contradiction was recently interpreted by Morag et al. (2020) as alternating magmatic and amagmatic spreading episodes.

Immediately after their formation, pillow lavas were locally covered by umbers (Mn- and Fe-rich mudstones) and radiolarian mudstones of the Perapedhi Formation (Robertson and Hudson 1973; Robertson and Fleet 1976; Robertson 1977; Blome and Irwin 1985; Bragina 2008). During the Campanian, volcanogenic sediments of the Kannaviou Formation were deposited in western and southern Cyprus, which were later extensively overlain by Maastrichtian to Oligocene deep-sea pelagic carbonates of the Lefkara Formation (Chen and Robertson 2019; Robertson and Hudson 1974). This deposition temporally overlaps with a 90° anti-clockwise Troodos microplate rotation between Campanian and Early Eocene (Clube et al. 1985; Morris et al. 1990).

As a consequence of subduction reactivation, serpentine diapirism, and initial collision with the Eratosthenes seamount, the Troodos oceanic crust experienced two stages of uplift: minor uplift from Late Oligocene to Early/Middle Miocene, and major uplift in Pleistocene (Robertson 1977, 1998; Kinnaird et al. 2011; Robertson et al. 2012; Main et al. 2016; Morag et al. 2016).

### Izu–Bonin forearc and rear arc

The Izu–Bonin trench in the Western Pacific is the geodynamic expression of the steep subduction of the Pacific Plate beneath the Philippine Sea Plate (e.g., Stern and Bloomer 1992; Bloomer et al. 1995). Subduction initiated ~ 52 Ma along a transform fault or fracture zone in the proto-forearc area where the dense Pacific Plate sank into the mantle (Reagan et al. 2019). This sinking caused asthenospheric upwelling accompanied by decompression melting in the forearc and rear arc where forearc basalts (FAB) and FAB-like rocks (~ 49–47 Ma) were emplaced, respectively (Brandl et al. 2017; Hickey-Vargas et al. 2018; Ishizuka et al. 2018; Reagan et al. 2019; Shervais et al. 2019). Within ~ 2 Myr after subduction initiation in the forearc, the melting mechanism changed from decompression to fluid-induced melting. Consequently, vesicle-rich porphyritic pillow lavas and massive sheet flows of boninitic composition were generated in the forearc (Reagan et al. 2017, 2019), but are absent in the rear arc (Arculus et al. 2015b). The spreading regime of the Izu–Bonin SSZ is poorly defined. Volcanic rock ages were used to propose intermediate to fast spreading rates (Ishizuka et al. 2018; Reagan et al. 2019).

Izu–Bonin forearc volcanism was followed by a > 15-Myr-long hiatus, which terminated in Oligocene when pelagic carbonates were deposited in normal fault-bounded grabens and basins (Robertson et al. 2018). Drill cores expose fault zones with various kinematic features including discrete normal, normal–oblique, and strike-slip faults as well as cataclastic shear zones down to a depth of ~ 535 m below seafloor (Site U1439). Extensional fractures and mineralized veins are related to these faults and fault zones (Kurz et al. 2019). These structures formed due to tectonic extension, which is explained by eastward slab rollback of the Pacific Plate (Faccenna et al. 2018; Kurz et al. 2019). Subsequent sedimentation from early Miocene to early Pliocene was characterized by the deposition of carbonate-poor hemipelagic clay and mud as well as tephra (Robertson et al. 2018). These sedimentary episodes temporally overlap with rifting of the Kyushu-Palau ridge, a remnant volcanic arc, and the formation of the Shikoku and Parece-Vela basins that separated the rear arc from the forearc between ~ 29 and ~ 15 Ma (Okino et al. 1998; Sdrolias et al. 2004). Thus, the rear arc crust translated into its present-day location in the Amami-Sankaku basin (Ishizuka et al. 2011). In contrast to the forearc, the igneous rear arc crust was covered by sedimentary deposits immediately after its formation (Arculus et al. 2015b). Possibly due to the central position of the Amami-Sankaku basin, no major faults were observed at Site U1438 and sediments are almost horizontally layered as seismic profiles show. Minor faults occur locally and are restricted to the sedimentary cover (Arculus et al. 2015a, b).

## Methods

### Collection of data

This study is primarily based on the data published in Quandt et al. (2018, 2019, 2020a) who attributed REE + Y concentrations and isotopic compositions (δ^18^O, δ^13^C, Δ_47_, ^87^Sr/^86^Sr) to specific vein types (Table 2). These data are complemented by published δ^18^O, δ^13^C, Δ_47_, and ^87^Sr/^86^Sr compositions (Alt et al. 1998; Gillis et al. 2015; Weinzierl et al. 2018; Coogan et al. 2019) that lack vein-type information. Previously unpublished electron microprobe element mappings extend the data set. Only data on secondary minerals from volcanic units are considered. The data from Quandt et al. (2018, 2019, 2020a) are archived at Pangaea Data Archiving & Publication (Quandt et al. 2020b, https://doi.pangaea.de/10.1594/PANGAEA.920681).

**Table 2:** References of data used in this study

	Quandt et al. (2018)	Quandt et al. (2019)	Gillis et al. (2015)	Coogan et al. (2019)	Weinzierl et al. (2018)	Quandt et al. (2020a)	Alt et al. (1998)	This study
Location	Troodos ophiolite	Troodos ophiolite	Troodos ophiolite	Troodos ophiolite	Troodos ophiolite	Izu–Bonin forearc/rear arc	Izu–Bonin forearc	All study areas
Vein type	X					X		
Fluid inclusion homogenization temperatures	X							
δ^13^C		X	X	X		X	X	
δ^18^O		X	X	X	X	X	X	
Δ_47_		X		X		X		
^87^Sr/^86^Sr		X	X	X	X	X		
REE + Y		X				X		
Element mappings								X

The geochemical data presented in Gillis et al. (2015) include data from Gillis (1987), Gillis and Robinson (1990), and Staudigel and Gillis (1990)

The Troodos geochemical data set presented here comprises samples from several pillow lava outcrops throughout the Troodos ophiolite (Gillis et al. 2015; Quandt et al. 2018; Weinzierl et al. 2018) as well as samples recovered during the Cyprus Crustal Study Project drill sites CY1 and CY1A (Gillis and Robinson 1990; Gillis et al. 2015). Samples from the Western Pacific Izu–Bonin forearc and rear arc were recovered from ODP (Ocean Drilling Program) Site 786 (Fryer et al. 1996) and IODP Sites U1438-U1441 (Arculus et al. 2015a; Reagan et al. 2015).

Mineralized veins from the Izu–Bonin forearc and rear arc are here considered as a single sample series that refers to as “Izu–Bonin forearc/rear arc” veins. Veins from the Izu–Bonin forearc and rear arc are hosted in genetically related ~ 52–47-Ma-old volcanic rocks that were juxtaposed until ~ 29 Ma (Reagan et al. 2017, 2019; Hickey-Vargas et al. 2018; Ishizuka et al. 2011, 2018). Considering that > 80% of secondary mineralization in oceanic crust is completed within ~ 25 Myr after crust formation (Coogan and Gillis 2018), this probably means that most veins pervading the rear arc crust formed prior to the separation of the rear arc from the forearc ~ 29 Ma (Okino et al. 1998; Sdrolias et al. 2004; Ishizuka et al. 2011).

Detailed descriptions of analytical methods are given in the respective publications. For consistency, δ^18^O formation temperatures of calcites were calculated using the calibration of Coplen (2007) and assuming equilibrium precipitation from seawater with a δ^18^O value of − 1‰ VSMOW. Conversely, clumped isotopes provide formation temperatures, which can be used to calculate the parental fluid δ^18^O value. Δ_47_ values of calcites were consistently converted to clumped isotope temperatures according to the inorganic calcite calibration of Daëron et al. (2019). Based on these temperatures and the calcite δ^18^O values, the parental fluid δ^18^O compositions were calculated using the calibration of Coplen (2007). Y anomalies are quantified by the Y/Ho ratio (Bau et al. 1997). Eu anomalies (Eu/Eu*_PAAS_) were calculated according to Eu/Eu*_PAAS_ = 2 × Eu_PAAS_/[Sm_PAAS_ + Gd_PAAS_] (Tostevin et al. 2016) and using Post-Archean Australian Shale (PAAS)-normalized REE + Y concentrations (McLennan 1989). Normal distributions were calculated according to $f\left( {x;\mu ,\sigma } \right) = \frac{1}{{\sqrt {2\pi \sigma^2 } }}e^{ - \left( {\frac{{\left( {x - \mu } \right)^2 }}{2\sigma^2 }} \right)} ,$ where *x* is the value for which the normal distribution is calculated, µ is the mean, and σ and σ^2^ are the standard deviation and variance, respectively.

### Electron microprobe element mappings

Electron microprobe element mappings of major (Ca) and trace elements (e.g., Mg, Mn, Fe, Na, Si) were acquired using a JEOL JXA 8200 electron microprobe equipped with five wavelength-dispersive spectrometers at the University of Leoben (Austria). Prior to analysis, polished vein thin sections were carbon coated. Measurements were conducted with an acceleration voltage of 15 kV and a beam current of 50–100 nA.

### Vein-type classification

Based on thin section and CL microscopy, mineralized veins were subdivided into blocky, antitaxial, transitional, completely and incompletely sealed syntaxial vein types, micrite-filled fractures, and mineralized vesicles (Quandt et al. 2018, 2019, 2020a). For reasons of clarity, we strictly adopt the vein-type classification after Bons et al. (2012) for this study considering only blocky, syntaxial, and antitaxial vein types. Hence, transitional veins marking the textural transition from syntaxial to antitaxial vein growth (Quandt et al. 2020a) are assigned to the antitaxial vein type. Similarly, vesicle calcites as well as late-stage calcites situated in cavities where vein sealing by early-stage quartz or analcime was incomplete (Quandt et al. 2018) are classified as blocky veins. Micrite-filled veins representing injection of remobilized calcareous sediment into fractures were rejected.

## Results

### Published petrographic and mineralogical observations and their implications in comparison

Mineralized veins pervading the volcanic units of the Troodos ophiolite and Izu–Bonin forearc/rear arc document post-magmatic fluid-mediated mineralization of dilatational sites such as fractures and faults and displacive mineral growth. These structures were classified as blocky, syntaxial, and antitaxial veins (Quandt et al. 2018, 2020a, b). At Sites U1439–U1441, mineralized veins are more abundant than at Site U1438. Troodos pillow lava outcrops show a high abundance of mineralized veins comparable with Izu–Bonin forearc Sites U1439–U1441.

#### Blocky veins

**Fig. 2:**
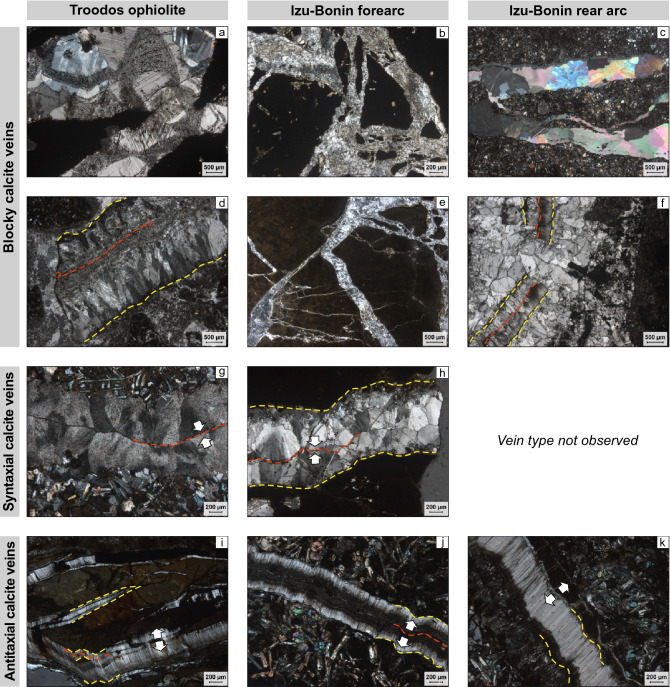
Representative blocky, syntaxial, and antitaxial calcite veins from the Troodos ophiolite, Izu–Bonin forearc, and Izu–Bonin rear arc. **a**–**f** Blocky vein calcites precipitated into fluid-filled fractures, which may be associated with rock brecciation. Host rock fragments may lack significant rotation, transport, rounding, and alteration, and, thus, display a jigsaw puzzle pattern. **d**, **f** Some blocky calcites enclose (slightly recrystallized) antitaxial vein fragments with intact vein margins (yellow dashed lines) and median lines (red dashed lines). **g**, **h** Syntaxial veins are characterized by inward growth of elongate-blocky calcites (white arrows) that exhibit growth competition. Fracture sealing occurs along a median line (red dashed line). **i**–**k** Antitaxial veins are composed of a median line (red dashed line) from which outward fibrous calcite growth displaced the host rock. Calcite fibers may connect parallel vein margins (yellow dashed line) and host rock fragments indicating the displacement direction. All photos were taken under crossed polarizers

**Fig. 3:**
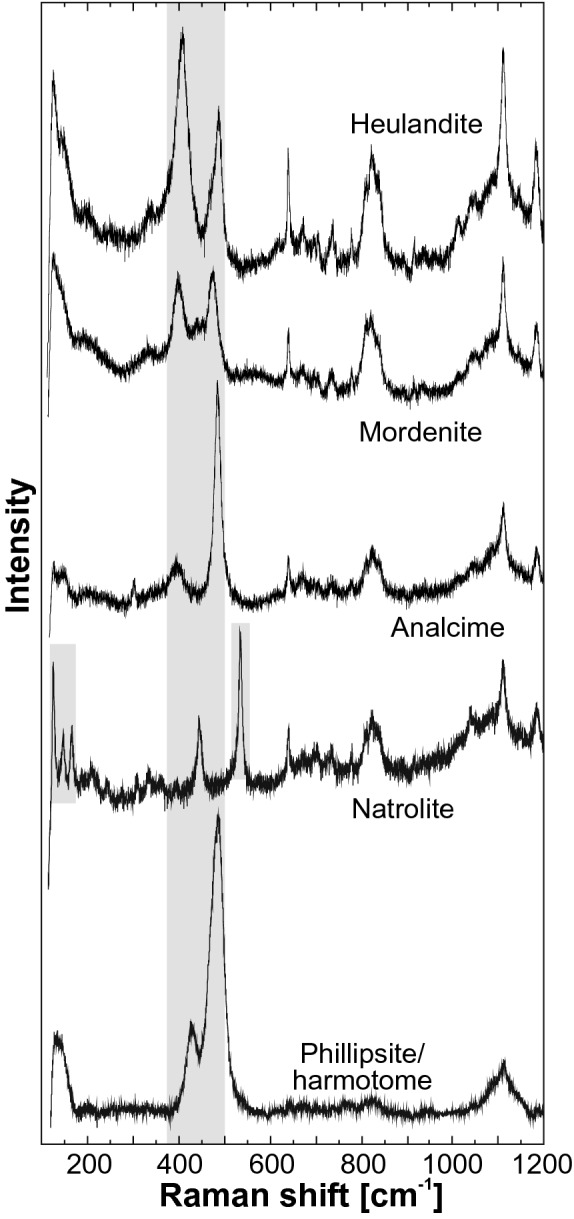
Representative Raman spectra of zeolites from the Troodos ophiolite and Izu–Bonin forearc. Based on indicative peak shifts (gray columns), zeolites are classified as heulandite (406, 486/cm), mordenite (396, 473/cm), analcime (383, 494/cm), natrolite (124, 145, 165, 443, 535/cm), and phillipsite/harmotome (420–430, 480–490/cm). The latter show nearly identical Raman spectra (Knight et al. 1989)

Thin section microscopy and Raman spectroscopy reveal that millimeters- to centimeters-thick irregular branching networks and millimeters-thick branchless structures filled with randomly distributed blocky calcite crystals are the dominant vein type in all study areas (Fig. 2a–f; Table 3; Quandt et al. 2018, 2020a). In the Troodos ophiolite, blocky calcites are frequently preceded by early-stage quartz and analcime (Fig. 3; Quandt et al. 2018) that are indicative of elevated to high formation temperatures (~ 75–300 °C; Chipera and Apps 2001). Blocky calcites in the Izu–Bonin forearc are in cases accompanied by argillaceous vein selvedges, phillipsite and/or harmotome (Fig. 3), and palagonite (Quandt et al. 2020a), which are interpreted as low-T (< 75 °C) precipitates and alteration products (Inoue 1995; Chipera and Apps 2001; Stroncik and Schmincke 2002). In all study areas, blocky calcites typically enclose host rock fragments and altered glass (palagonite) shards (Quandt et al. 2018, 2020a). In some cases, blocky calcites cement intact antitaxial vein fragments. These features indicate host rock fracturing and brecciation, which possibly propagated along zones of weakness such as pre-existing veins. They also suggest advective fluid flow and growth into fluid-filled fractures under ongoing nucleation of new crystals (Bons et al. 2012; Quandt et al. 2018, 2020a). Especially in the Izu–Bonin forearc, host rock fragments lack significant rotation, transport, rounding, and alteration, and thus display a jigsaw puzzle pattern (Fig. 2b, e). Such patterns are less obvious in blocky veins from the Troodos ophiolite. Independent of the location, blocky veins may be associated with alteration halos (Quandt et al. 2018, 2020a). CL microscopy reveals that most vein calcites show simple growth zonations that are characterized by non-luminescent cores and luminescent grain boundaries (Quandt et al. 2018, 2020a). More complex, highly oscillatory growth zonations with (intra-) sectoral zones are restricted to the Troodos ophiolite and indicate geochemical self-organization (Quandt et al. 2018).

**Table 3:** Summary of calcite vein petrography and genetic implications

	Troodos ophiolite			Izu–Bonin forearc			Izu–Bonin rear arc	
Vein type (percentage)	Blocky (75)	Syntaxial (15)	Antitaxial (10)	Blocky (85)	Syntaxial (5)	Antitaxial (10)	Blocky (60)	Antitaxial (40)
Vein macro-structures	Millimeters–centimeters-thick vein networks and branchless veins	Millimeters-thick branchless veins	Millimeters–centimeters-thick crosscutting vein networks	Millimeters–centimeters-thick vein networks and branchless veins	Millimeters-thick branchless veins	Millimeters-thick branchless veins	Millimeters-thick vein networks and branchless veins	Millimeters-thick branchless veins
Vein mineralogy (percentage)	Calcite (65), quartz (20), analcime (15), natrolite (trace amounts)	Calcite (60), mordenite and heulandite (15), quartz (15), analcime (10)	Calcite (100), clay minerals (trace amounts)	Calcite (90), phillipsite/ harmotome (10), clay minerals and palagonite (trace amounts)	Calcite (95), phillipsite/ harmotome (5)	Calcite (100), clay minerals (trace amounts)	Calcite (100)	Calcite (100), clay minerals (trace amounts)
Mode of rock failure	Extensional and/or hydro-fracturing	Extensional fracturing	Displacive mineral growth due to crystallization pressure	Extensional and hydro-fracturing	Extensional fracturing	Displacive mineral growth due to crystallization pressure	Extensional and/or hydro-fracturing	Displacive mineral growth due to crystallization pressure
Mode of fluid flow	Advection	Advection	Diffusion	Advection	Advection	Diffusion	Advection	Diffusion
Vein mineral growth dynamics	Ongoing nucleation of new crystals, no systematic growth direction	Inward mineral growth, sealing along a median line, inhibited nucleation of new crystals, growth competition	Outward extension of existing crystals from a median line	Ongoing nucleation of new crystals, no systematic growth direction	Inward mineral growth, sealing along a median line, inhibited nucleation of new crystals, growth competition	Outward extension of existing crystals from a median line	Ongoing nucleation of new crystals, no systematic growth direction	Outward extension of existing crystals from a median line
Rate of fracture opening (F) to mineral growth (M)	F > M (Growth within fluid-filled space)	F = M (crack and sealing)	F < M (displacive mineral growth)	F > M (Growth within fluid-filled space)	F = M (crack and sealing)	F < M (displacive mineral growth)	F > M (Growth within fluid-filled space)	F < M (displacive mineral growth)
Crystal habit (length–width ratio)	Blocky (Not applicable)	Elongate-blocky (< 10)	Fibrous (≫ 10)	Blocky (Not applicable)	Elongate-blocky (< 10)	Fibrous (≥ 10)	Blocky (Not applicable)	Fibrous (≫ 10)

Data originate from Quandt et al. (2018, 2019, 2020a)

#### Syntaxial veins

Syntaxial veins are millimeters-thick, usually branchless structures with parallel vein margins. They are characterized by a median line and growth competition between inwardly growing elongate-blocky crystals (Fig. 2g, h, Table 3; Quandt et al. 2018, 2020a). These microtextures are particularly well developed in the Troodos ophiolite and resulted from similar rates of mineral growth and fracture opening during which the nucleation of new crystals was suppressed (Fisher and Brantley 1992; Hilgers et al. 2001; Bons et al. 2012). Together with the lack of solid inclusion bands, syntaxial veins indicate onefold crack and sealing after advective fluid circulation through extensional fractures. In the Troodos ophiolite, syntaxial veins consist of calcite, analcime, mordenite and heulandite, or quartz (Quandt et al. 2018; Fig. 3). Excluding calcite, this mineral assemblage indicates elevated to high formation temperatures between ~ 75 and ~ 300 °C (Chipera and Apps 2001). Syntaxial veins in the Izu–Bonin forearc are solely composed of calcite. In the Izu–Bonin rear arc, syntaxial veins are absent (Quandt et al. 2020a). CL microscopy reveals that elongate-blocky calcites from the Troodos ophiolite show a high, homogeneously distributed CL; whereas, elongate-blocky calcites from the Izu–Bonin forearc are characterized by non-luminescent cores and luminescent grain boundaries (Quandt et al. 2018, 2020a).

#### Antitaxial veins

Antitaxial veins occur in all study areas and consist of fibrous calcites oriented perpendicular to the wall rock (Quandt et al. 2018, 2020a; Fig. 2i–k, Table 3). Antitaxial veining initiated at one vein margin (unitaxial) or along a median line from which outward calcite fiber growth displaced the host rock. Most studies suggest that antitaxial veining is fluid diffusion fed and crystallization pressure driven (Watts 1978; Means and Li 2001; Wiltschko and Morse 2001; Elburg et al. 2002; Meng et al. 2018, 2019). This means that the rate of fibrous calcite growth exceeded the rate of vein opening equivalent to veining without fracturing (Hilgers et al. 2001). During their growth, fibers incorporated multiple wallrock-parallel solid inclusion bands and trails. In the Troodos ophiolite, antitaxial veins are restricted to a single pillow lava outcrop where they constitute centimeters-thick branching and crosscutting networks hosting rock fragments (Quandt et al. 2018). Antitaxial veins in the Izu–Bonin forearc/rear arc are millimeters-thick branchless structures with argillaceous selvedges and free of host rock fragments. They are either subordinate (forearc) or common features (rear arc) that approximate the abundance of blocky veins (Quandt et al. 2020a). Independent of the location and macrostructure, CL microscopy reveals a decrease in CL intensity in fiber growth direction and multiple median line-parallel cathodoluminescent bands (Quandt et al. 2018, 2020a). Where straight fibers connect corresponding vein markers on both sides of the vein, the displacement trajectory may be tracked (Durney and Ramsay 1973; Ramsay and Huber 1983; Bons et al. 2012). Similarly, angular and non-rotated host rock fragments may be easily pieced together. Curved fibers, particularly in antitaxial veins from the Troodos ophiolite, complicate tracking of the displacement trajectory (Quandt et al. 2019).

#### Fluid inclusions

**Fig. 4:**
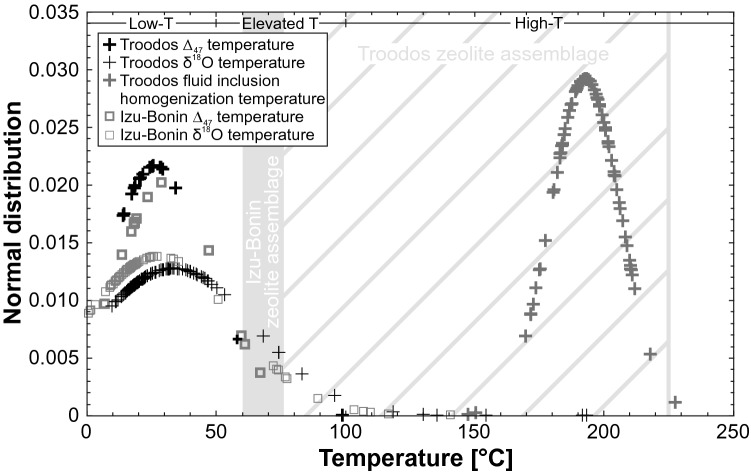
Summary of formation temperatures of vein and vesicle minerals from the Troodos ophiolite and Izu–Bonin forearc/rear arc inferred from fluid inclusion (calcite, quartz, analcime; Quandt et al. 2018), δ^18^O (calcite; Gillis and Robinson 1990; Gillis et al. 2015; Weinzierl et al. 2018; Quandt et al. 2019, 2020a, b), and Δ_47_ thermometry (calcite; Coogan et al. 2019; Quandt et al. 2019, 2020a, b). Site-specific zeolite assemblages define distinct temperature ranges (Chipera and Apps 2001)

Well-preserved fluid inclusions exclusively occur in localized blocky calcite, quartz, and analcime veins and vesicles from the Troodos ophiolite. They consist of two phases (liquid and vapor) and were entrapped during mineral growth (primary) or healing of microfractures (secondary; Quandt et al. 2018). Isobaric cooling caused re-equilibration and decrepitation of large primary fluid inclusions hosted in Mn-rich domains of blocky and syntaxial vein calcites (Quandt et al. 2018). Microthermometry of primary two-phase fluid inclusions yielded seawater-like salinities and minimum entrapment temperatures (homogenization temperatures) that were constrained by hydrostatic pressure estimates resulting in precipitation temperatures between 140 and 230 °C (Fig. 4; Quandt et al. 2018).

### Published geochemical compositions and their implications in comparison

#### Stable isotopes

**Fig. 5:**
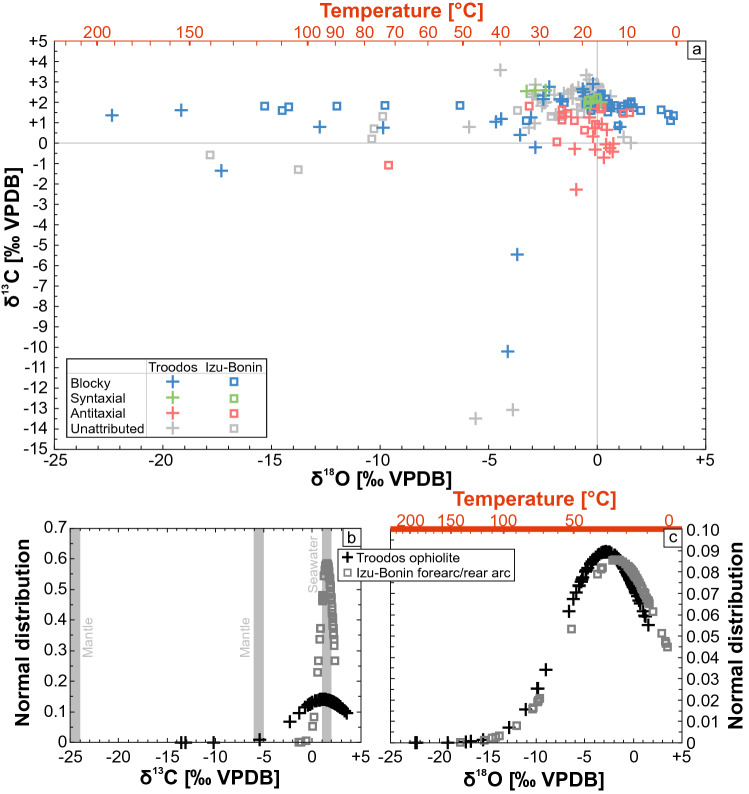
**a** δ^13^C and δ^18^O values of vein calcites from the Troodos ophiolite (Gillis et al. 2015; Weinzierl et al. 2018; Coogan et al. 2019; Quandt et al. 2019) and Izu–Bonin forearc/rear arc (Alt et al. 1998; Quandt et al. 2020a, b). **b**, **c** δ^13^C and δ^18^O normal distributions. Mantle-derived δ^13^C shows a bimodal distribution with peaks at − 5 and − 25‰ VPDB (Deines 2002). Temperature axes are constructed using the calibration of Coplen (2007) assuming precipitation from seawater with a δ^18^O average value of − 1‰ VSMOW

The secondary calcites from the Troodos ophiolite and Izu–Bonin forearc/rear arc show a similar δ^18^O distribution pattern that is composed of a dense point cluster between + 4 and − 4‰ VPDB from which blocky veins trend toward significantly negative δ^18^O values up to − 23‰ VPDB (Fig. 5a). This is supported by the δ^18^O normal distributions of the respective study areas (Fig. 5c) in which the mean δ^18^O values of the sample suites are − 2.8‰ (Troodos) and − 1.8‰ VPDB (Izu–Bonin). Both normal distributions show similar standard deviations resulting in similar curve shapes that are offset by ~ 1‰ VPDB. Corresponding calcite δ^18^O formation temperatures fall within the range of 0–30 °C (Izu–Bonin) and 10–50 °C (Troodos), respectively (Fig. 4). Elevated (up to 100 °C) and high formation temperatures (> 100 °C) are localized features of samples situated in the extrusive–dyke transition zone of the respective study areas (Quandt et al. 2019, 2020a). However, negative δ^18^O values may also result from low-T fluid–rock interaction (Quandt et al. 2020a).

In contrast to δ^18^O, the δ^13^C distribution patterns of secondary calcite from the Troodos ophiolite and Izu–Bonin forearc/rear arc differ. Secondary calcites from the Izu–Bonin forearc/rear arc mainly fall within the narrow range of + 1 to + 2‰ VPDB (Fig. 5a). Troodos pillow lava-hosted secondary calcites show a wider δ^13^C range due to a trend toward negative δ^13^C compositions up to − 13.5‰ VPDB. Variably negative δ^13^C points to different degrees of input from the mantle carbon reservoir (Quandt et al. 2019; Fig. 5b). Independent of the location, antitaxial vein calcites have consistently lower δ^13^C values (≤ + 1.8‰ VPDB) than syntaxial veins (≥ + 1.9‰ VPDB) and lower average δ^13^C values (+ 0.6‰ VPDB) than blocky veins (+ 1.4‰ VPDB).

#### Clumped isotopes

**Fig. 6:**
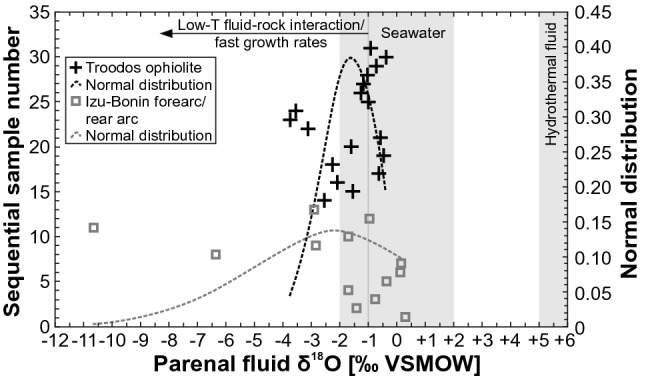
Clumped isotope-derived parental fluid δ^18^O values and normal distributions of vein calcites from the Troodos ophiolite (Coogan et al. 2019; Quandt et al. 2019) and Izu–Bonin forearc/rear arc (Quandt et al. 2020a, b). Reference values of seawater and hydrothermal fluid are taken from Hoefs (2015)

Clumped isotope temperatures of most vein calcites from both study areas are < 30 °C (Fig. 4) and only a few vein calcites show higher clumped isotope temperatures up to ~ 100 °C. Hence, vein calcites from both study areas show similar mean clumped isotope temperatures in agreement with mean δ^18^O temperatures. Corresponding parental fluid δ^18^O compositions fall predominantly within or close to the conservatively estimated seawater range between − 2 and + 2‰ VSMOW (Fig. 6). A few significantly lowered parental fluid δ^18^O values < − 3‰ VSMOW were interpreted as the result of low-T fluid–rock interaction (Quandt et al. 2020a) and disequilibrium precipitation due to fast growth rates (Coogan et al. 2019), respectively. Mean parental fluid δ^18^O values of vein calcites from the Troodos ophiolite (− 1.6‰ VSMOW) and Izu–Bonin forearc/rear arc areas (− 1.5‰ VSMOW) excluding δ^18^O values < − 10‰ VSMOW fall within the seawater range.

#### Rare earth elements and yttrium

**Fig. 7:**
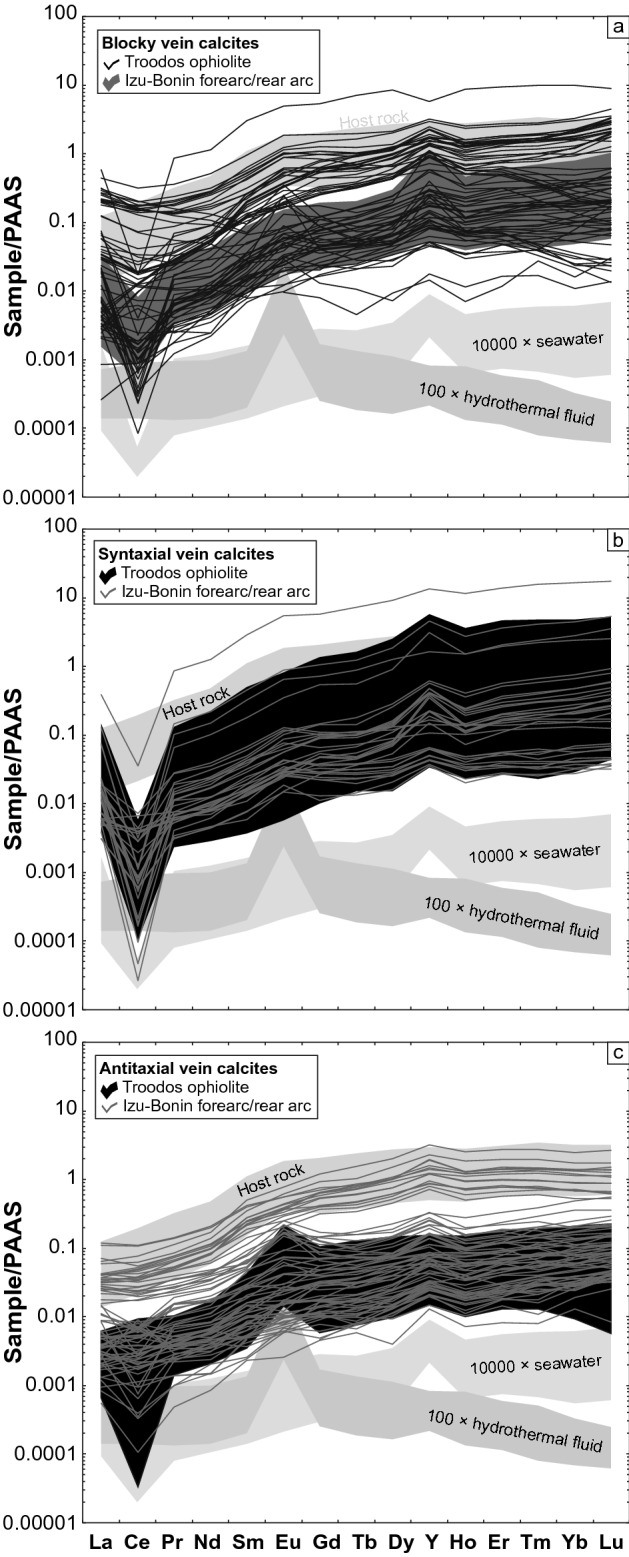
PAAS-normalized (McLennan 1989) REE + Y distribution patterns of blocky (**a**), syntaxial (**b**), and antitaxial vein calcites (**c**) from the Troodos ophiolite and Izu–Bonin forearc/rear arc. The samples are compared to host rocks (Regelous et al. 2014; Shervais et al. 2019), hydrothermal fluids (Bau and Dulski 1999), and seawater (Zhang and Nozaki 1996)

Vein calcites from the Troodos ophiolite and Izu–Bonin forearc/rear arc show heavy rare earth element-enriched PAAS-normalized (McLennan 1989) REE + Y distribution patterns. Independent of the study area, the REE + Y distribution patterns reveal consistent vein type-specific Ce, Eu, and Y anomalies (Fig. 7). Syntaxial vein calcites show uniform seawater-like distribution patterns with negative Ce anomalies and positive Y anomalies. Antitaxial vein calcites are characterized by high proportions of positive Eu, positive Ce, and reduced Y anomalies relative to seawater. These REE + Y characteristics are indicative of hydrothermal fluids. Blocky vein calcites display variable Ce, Eu, and Y anomalies. Seawater-like negative Ce and positive Y anomalies resemble the distribution patterns of syntaxial vein calcites; whereas, reduced Y and elevated Eu anomalies relative to seawater are typical for fluid–rock interaction and hydrothermal fluids, respectively.

**Fig. 8:**
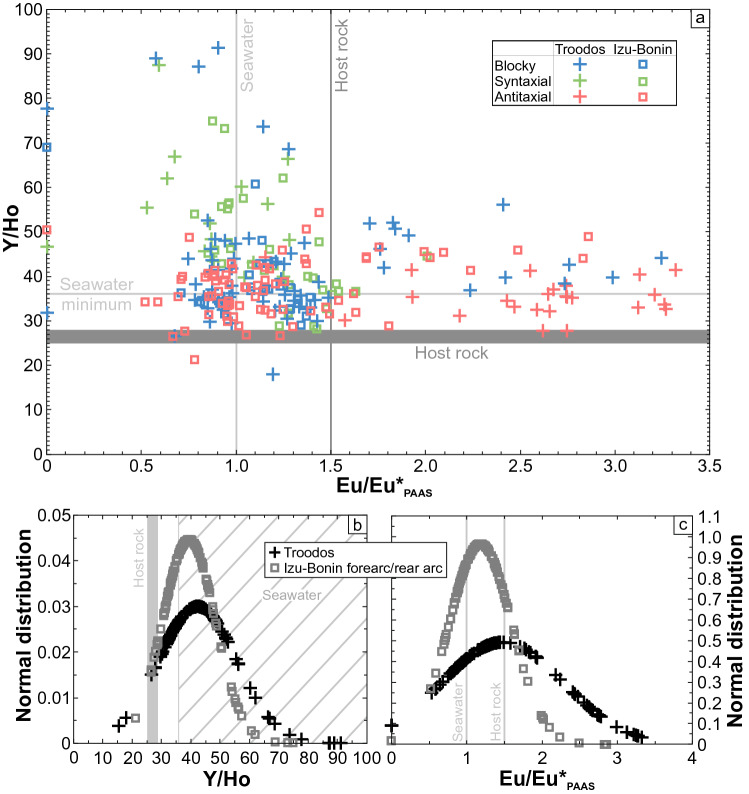
Calculated Eu/Eu*_PAAS_ and Y/Ho ratios of blocky, syntaxial, and antitaxial vein calcites from the Troodos ophiolite and Izu–Bonin forearc/rear arc. The samples are compared to host rocks (Regelous et al. 2014; Shervais et al. 2019), hydrothermal fluids (Bau and Dulski 1999), and seawater (Zhang and Nozaki 1996)

These observations are supported by calculated Eu and Y anomalies (Fig. 8a), which suggest that the different vein growth dynamics such as precipitation in fluid-filled fractures (blocky), crack and sealing (syntaxial), and diffusion-fed and crystallization-driven displacive fiber growth (antitaxial) are crucial factors for the REE + Y geochemistry of vein calcites (Quandt et al. 2019, 2020a). Syntaxial vein calcites from all study areas are predominantly characterized by varying seawater-like Y/Ho ratios (36–87) and seawater-like Eu/Eu*_PAAS_ (0.5–1.5). In contrast, antitaxial vein calcites from all study areas show reduced Y/Ho ratios (< 50) and increased Eu/Eu*_PAAS_ (up to 3.4) relative to seawater. Blocky vein calcites show a high variation in Y/Ho ratios (18–91) and Eu/Eu*_PAAS_ (0.6–3.2) and, thus, overlap with syntaxial and antitaxial vein calcite fields. This compositional overlap is reflected by the normal distributions of Y/Ho and Eu/Eu*_PAAS_ of each study area (Fig. 8b, c). Thus, mean Y/Ho ratios (42 and 39) fall within the lower range of seawater (> 36) and mean Eu/Eu*_PAAS_ (1.2 and 1.5) are seawater-like. However, Troodos pillow lava-hosted vein calcites show a relatively high proportion of Eu/Eu*_PAAS_ exceeding the Eu/Eu*_PAAS_ range of seawater (up to 1.5). Besides antitaxial vein calcites that acquired their high Eu/Eu*_PAAS_ during fluid interaction with hydrothermal sediments (Quandt et al. 2019), blocky vein calcites hosting high-T fluid inclusions also show high Eu/Eu*_PAAS_ pointing to a temperature dependence of the positive Eu anomaly as proposed in previous studies (Sverjensky 1984; Bau 1991; Danielson et al. 1992; Allen and Seyfried 2005; Bau et al. 2010).

#### Radiogenic strontium isotopes

**Fig. 9:**
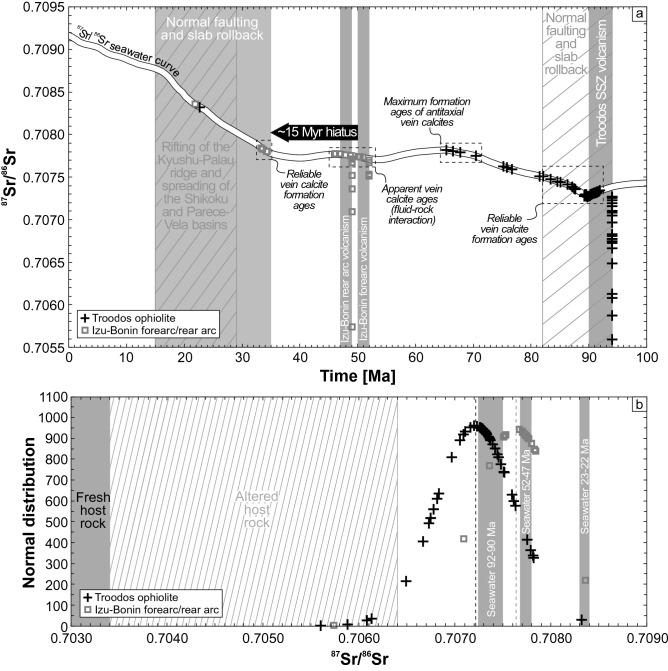
**a** ^87^Sr/^86^Sr stratigraphy of vein calcites from the Troodos ophiolite (Coogan et al. 2019; Gillis et al. 2015; Quandt et al. 2019; Weinzierl et al. 2018) and Izu–Bonin forearc/rear arc (Quandt et al. 2020a, b) using the ^87^Sr/^86^Sr seawater curve (McArthur et al. 2001). Vein calcites with seawater signatures inferred from REE + Y, δ^18^O, δ^13^C, and Δ_47_ isotope geochemistry give precipitation ages between ~ 92 and ~ 82 Ma (Troodos ophiolite), and between ~ 35 and ~ 33 Ma (Izu–Bonin forearc). Where calcites intersect the ^87^Sr/^86^Sr seawater curve multiple times or lack intersections, only the respective maximum ages are plotted. **b** Normal distributions of ^87^Sr/^86^Sr of vein calcites from the Troodos ophiolite and Izu–Bonin forearc/rear arc show mean ^87^Sr/^86^Sr values that are slightly lower than the seawater ^87^Sr/^86^Sr value at the respective time of SSZ volcanism. See text for references of geological background information (gray columns and shaded boxes)

Compared with the other geochemical tracers, ^87^Sr/^86^Sr values are less conclusive due to changing ^87^Sr/^86^Sr seawater ratios over time. In most cases, ^87^Sr/^86^Sr ratios alone cannot prove precipitation from seawater but in turn can give evidence of fluid–rock interaction. Collected ^87^Sr/^86^Sr values of vein calcites are displayed in Fig. 9 relative to the ^87^Sr/^86^Sr seawater curve (McArthur et al. 2001). Except for vein calcites from the Izu–Bonin rear arc that consistently have lower ^87^Sr/^86^Sr ratios than seawater, most vein calcites intersect the ^87^Sr/^86^Sr seawater curve. These intersections may be considered as an estimate on the time of calcite precipitation (here referred to as intersection age), provided that the calcites precipitated from pristine seawater and preserved its original ^87^Sr/^86^Sr composition (e.g., Hart and Staudigel 1978). The high sensitivity of the REE + Y to fluid–rock interaction compared with isotopic tracers makes them to a suitable tool to test if the calcites precipitated from pristine seawater. Indeed, REE + Y characteristics indicate that some vein calcites whose ^87^Sr/^86^Sr ratios intersect the ^87^Sr/^86^Sr seawater curve are affected by fluid–rock interaction probably involving Sr exchange between fluid and rock (Quandt et al. 2019, 2020a). Therefore, relative dating of calcites whose ^87^Sr/^86^Sr ratios intersect the ^87^Sr/^86^Sr seawater curve is limited to calcites with pristine REE + Y seawater signatures. This applies to syntaxial calcites in general as well as some blocky calcites from the Troodos ophiolite and Izu–Bonin forearc. As a result, only intersections with the Sr isotope seawater curve between ~ 94 and ~ 82 Ma (Troodos ophiolite) and between ~ 35 and ~ 33 Ma (Izu–Bonin forearc) represent reliable precipitation ages (Quandt et al. 2019, 2020a). Thus, major vein calcite precipitation in the Troodos SSZ initiated contemporaneously with volcanic activity and major normal faulting and persisted for ~ 10–20 Myr as also proposed by Gillis et al. (2015) and Coogan et al. (2019). In contrast, reliable ages of vein calcites from the Izu–Bonin forearc indicate vein formation > 15 Myr after volcanic activity following a hiatus (Robertson et al. 2018; Kurz et al. 2019). Vein calcites with ^87^Sr/^86^Sr ratios lacking intersections with the ^87^Sr/^86^Sr seawater curve including a high proportion of antitaxial calcites from the Izu–Bonin rear arc may have formed at any time after respective host rock formation. Even seawater-dominated fluid mixtures (99% seawater, 1% basalt) have significantly reduced ^87^Sr/^86^Sr ratios compared to pristine seawater (Quandt et al. 2020a). Concerning the samples from the Izu–Bonin forearc, this implies that calcites showing geochemical signatures indicative of weak fluid-rock interaction and intersecting the ^87^Sr/^86^Sr seawater curve between 52 and 46 Ma are probably 20 Myr younger than their intersections ages suggest. This is due to consistently increasing ^87^Sr/^86^Sr seawater ratios since ~ 40 Ma. Antitaxial calcites from the Troodos ophiolite show ^87^Sr/^86^Sr ratios that intersect the ^87^Sr/^86^Sr seawater curve but their REE + Y characteristics indicate fluid-rock interaction. Therefore, intersections represent maximum precipitation ages due to mainly increasing seawater ^87^Sr/^86^Sr ratios (Quandt et al. 2019). The normal distributions of ^87^Sr/^86^Sr ratios of vein calcites from both study areas are similarly characterized by mean ^87^Sr/^86^Sr ratios that are slightly lower than the ^87^Sr/^86^Sr seawater ratio at the time of respective SSZ volcanism (Fig. 9).

**Fig. 10:**
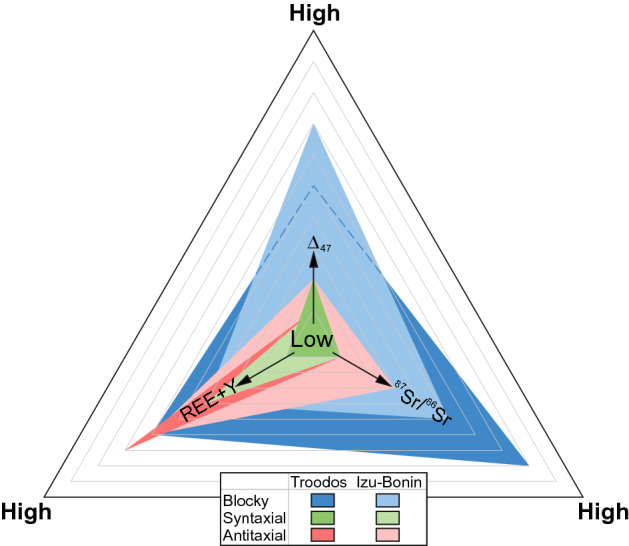
Summary of the relative extent of fluid–rock interaction for the different calcite vein types from the Troodos ophiolite and Izu–Bonin forearc/rear arc based on different geochemical tracers (REE + Y, ^87^Sr/^86^Sr, Δ_47_). Each bisector of an angle corresponds to a geochemical tracer and indicates increasing fluid-rock interaction from the incenter (pristine seawater) to each angle (hydrothermal fluid). For instance, antitaxial vein calcites from both study areas show acute triangles with acute angles pointing along the REE + Y bisector, whereas the obtuse angles point along the ^87^Sr/^86^Sr and Δ_47_ bisectors. This probably indicates that the REE + Y are more sensitive to fluid–rock interaction than ^87^Sr/^86^Sr and Δ_47_

The parental fluid signatures inferred from ^87^Sr/^86^Sr, REE + Y, and Δ_47_ data are compared in Fig. 10 for the different vein types. In this ternary diagram, each bisector of an angle corresponds to an indicative geochemical tracer axis (^87^Sr/^86^Sr, REE + Y, Δ_47_). Along each bisector of an angle, the relative degree of fluid–rock interaction increases from the incenter (pristine seawater) to the angle (hydrothermal fluid). Among blocky and antitaxial vein calcites, compositional ranges from seawater to hydrothermal fluids are common. The resulting triangles are larger than the triangles for syntaxial vein calcites, which are consistently characterized by relatively pristine seawater signatures.

### New element mappings

**Fig. 11:**
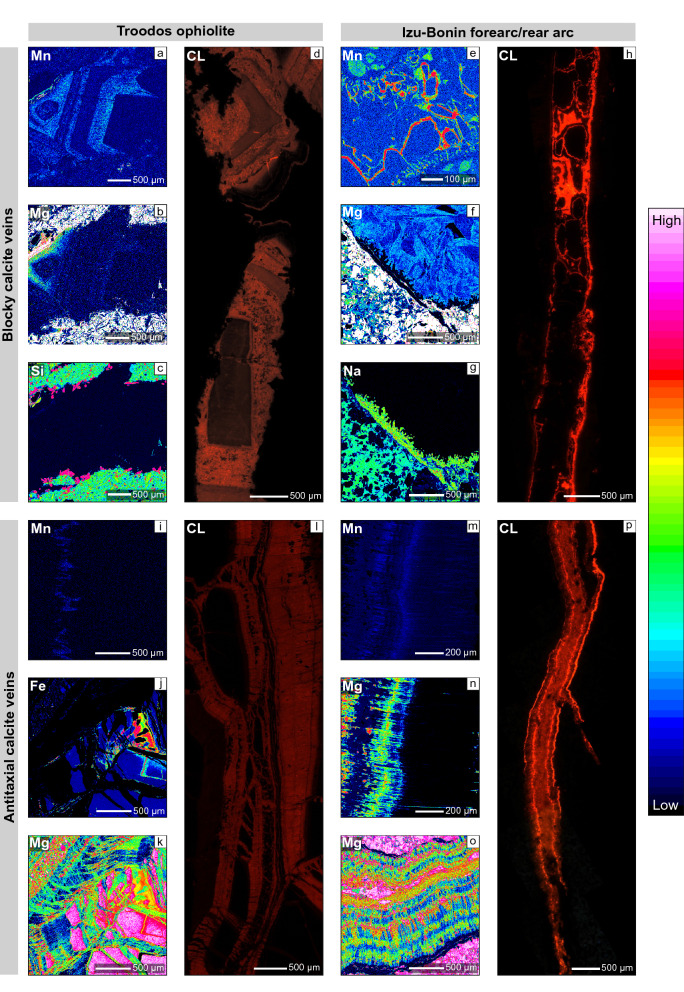
Microprobe element mappings and CL of selected vein calcites. **a**–**d** Complex growth-zoned (Mn and Mg) high-T blocky vein calcites (Troodos ophiolite) with early-stage quartz indicated by high Si concentrations. The element mappings (**a**–**c**) represent the same area and belong to the sample shown in (**d**). **e**–**h** Simple growth-zoned (Mn) low-T blocky vein calcites (Izu–Bonin forearc/rear arc) with feathery Mg distribution. High Na concentrations indicate phillipsite/harmotome. Mg and Na mappings (**f** and **g**) represent the same area. **i**–**p** Antitaxial vein calcites from the Troodos ophiolite and Izu–Bonin forearc/rear arc show Mn and Mg bands perpendicular to fiber growth direction. Mn bands confirm CL observations. Mg bands extend in fiber growth direction and coincide with host rock inclusion bands and trails. In the Troodos ophiolite, antitaxial veins constitute branching networks enclosing host rock fragments, which can be easily pieced together. Fe and Mg (**j** and **k**) mappings represent the same area and belong to the sample shown in (**l**). Mn and Mg (**m** and **n**) show the same area

Element mappings confirm observations from CL microscopy (Fig. 11; Quandt et al. 2018, 2020a). Thus, simple and complex cathodoluminescent growth zonations of blocky calcites from all study areas are based on increased Mn concentrations. In some cases, increased Mn concentrations are accompanied by Mg enrichments and Ca depletions indicating elemental substitution. However, most blocky, syntaxial, and antitaxial calcites from the Troodos ophiolite and Izu–Bonin forearc/rear arc show relatively homogeneous Ca distributions. Si and Na mappings highlight the occurrence of early-stage quartz and phillipsite/harmotome in blocky calcite veins from the Troodos ophiolite and Izu–Bonin forearc, respectively.

Striking differences between the different locations involve the Mg distribution in blocky calcites (Fig. 11). In the Izu–Bonin forearc/rear arc, blocky calcites are characterized by feathery Mg distributions, whereas blocky calcites from the Troodos ophiolite show homogenous Mg distributions.

Antitaxial vein calcites (Fig. 11) from the different study areas are characterized by similar Mn and Mg distributions patterns. In agreement with CL microscopy, Mn is concentrated along bands perpendicular to the fiber growth directions (Quandt et al. 2018, 2020a). Mg is similarly distributed along bands but rather extends in fiber growth direction and coincides with host rock inclusion bands and trails. Hence, Mn and Mg bands are not congruent. Moreover, element mappings of Troodos pillow lava-hosted antitaxial calcites highlight how multiple, crosscutting fibrous veinlets with identifiable median lines displaced and brecciated the host rock, which may be easily pieced together. In cases, margins of host rock fragments are depleted in Mg, Fe, Mn indicating leaching processes.

## Discussion

### The development of vein microtextures and geochemical signatures

#### Blocky veins

This comparative analysis shows that the veins pervading the volcanic units of the Troodos ophiolite and Izu–Bonin forearc/rear arc share structural, microtextural, mineralogical, and vein type-specific geochemical characteristics. In all study areas, blocky calcite veins are the major vein type. The dominance of comparatively thick blocky calcite veins, which are consistently interpreted as the result of precipitation into fluid-filled fractures, implies substantial fracture-controlled advective fluid flow accompanied by high water–rock ratios in all study areas. This explains the predominance of seawater-dominated signatures (pristine to slightly modified seawater) and precipitation temperatures < 50 °C of most vein calcites from all study areas. Where calcite precipitation into fractures was delayed, fluid residence times increased and intensified fluid–rock interaction. This is demonstrated by argillaceous vein selvedges, vein-parallel alteration halos, early-stage euhedral quartz or zeolite growth along vein margins, and blocky calcites with reduced Y/Ho and ^87^Sr/^86^Sr ratios below respective seawater values.

At temperatures > 100 °C, blocky calcites developed Mn-controlled oscillatory growth zonations with (intra-) sectoral zones. Growth zonations of low-T blocky calcites are by far less complex. Hence, high-T conditions might be an important requirement for the development of oscillatory growth zonations. These patterns point to disequilibrium precipitation in a closed system in which the zonation develops without external input (e.g., Ortoleva et al. 1987; Reeder et al. 1990; Wang and Merino 1992).

The incorporation of antitaxial vein fragments into blocky veins and crosscutting relationships in all study areas suggest that fracturing is a recurring process, preferentially along zones of weakness such as pre-existing veins, which overprinted and destroyed original structures. Fracturing may be triggered by extensional tectonics, as described for the Izu–Bonin forearc (Kurz et al. 2019), thermal contraction, and/or fluid overpressure (Quandt et al. 2019, 2020a).

#### Syntaxial veins

Syntaxial vein microtextures, similar to those observed among veins from the Troodos ophiolite and Izu–Bonin forearc, were created by crack and sealing in numerical simulations (Hilgers et al. 2001). Hence, the naturally occurring syntaxial veins from the Troodos ophiolite and Izu–Bonin forearc imply fracture sealing by mineral growth simultaneously with fracture opening. Thus, fast fracture opening is accompanied by fast mineral growth, which may explain several structural and geochemical characteristics of the syntaxial vein calcites analyzed in this study. Although syntaxial vein calcites from the Troodos ophiolite formed at temperatures < 50 °C, they entrapped two-phase primary fluid inclusions that later re-equilibrated and decrepitated due to isobaric cooling (Quandt et al. 2018). Fluid inclusions usually do not nucleate vapor bubbles at temperatures < 50 °C (Pagel et al. 2018). However, the fast growth rates of syntaxial vein calcites probably provoked entrapment of particularly large two-phase fluid inclusions (Sisson et al. 1993). Furthermore, fast mineral growth rates reduce the fluid residence time hampering the extent of fluid–rock interaction. As a result, syntaxial calcites show pristine geochemical seawater signatures. However, due to fast growth rates, growth rate-sensitive proxies may not yield representative compositions.

#### Antitaxial veins

In contrast to facture-controlled blocky and syntaxial veining, antitaxial veins are fed by fluid diffusion and driven by the crystallization pressure of calcite fibers (Watts 1978; Means and Li 2001; Wiltschko and Morse 2001; Elburg et al. 2002; Meng et al. 2018, 2019). This means that the fiber growth rate exceeded the fracture opening rate (Hilgers et al. 2001) that possibly approximated zero equivalent to fracture-independent veining. Host rock displacement may be tracked where fibers connect corresponding vein markers and parallel vein margins. Moreover, multiple median line-parallel Mn-rich bands display incremental fiber growth stages (Urai et al. 1991; Hilgers and Sindern 2005).

Relative dating using ^87^Sr/^86^Sr stratigraphy of Troodos pillow lava-hosted fibrous vein calcites suggest that antitaxial veins formed after blocky veins; whereas, antitaxial vein fragments incorporated into blocky veins prove the opposite. This indicates that antitaxial veining may occur over an extended time range compared to blocky veining. Similar conclusions were made on fibrous veins sampled from drill cores recovered from the eastern Pacific crust (Alt et al. 1993, 1996; Harper and Tartarotti 1996; Tartarotti et al. 1996). These veins predominantly crosscut blocky veins indicating late-stage formation but are subordinately also crosscut by blocky veins. Therefore, the extent and time of antitaxial veining probably depends on the local geological environment involving high sedimentation rates, the absence of extensional fractures, and pervasive rock alteration. These processes contribute to hydraulic sealing of the oceanic crust and lower its permeability. Hydraulic sealing becomes more important with ongoing time implying a change from advective to diffusive fluid flow. As a result, diffusion-fed antitaxial veining gains in importance relative to blocky veining with crustal aging.

Fluid diffusion probably intensified fluid–rock interaction and lowered the Y/Ho and ^87^Sr/^86^Sr ratios of the fluid whose signature is recorded in antitaxial calcite fibers. The positive Eu anomalies commonly observed among antitaxial fibrous calcites are not directly related to high precipitation temperatures as indicated by low precipitation temperatures inferred from oxygen and clumped isotope thermometry. Instead, positive Eu anomalies of antitaxial calcite fibers may be associated with hydrothermal fluids that preserved their positive Eu anomalies after cooling (Bau et al. 2010) or interaction with hydrothermal sediments (Quandt et al. 2019).

### The influence of the local geological setting on veining: comparison between veining in the Troodos ophiolite and Izu–Bonin forearc/rear arc

Besides structural, microtextural, mineralogical, and geochemical similarities, the time of major calcite veining relative to crust formation in the respective study areas differs. This probably explains further differences such as higher peak formation temperatures up to 230 °C of vein and vesicle minerals from the Troodos ophiolite inferred from fluid inclusion microthermometry, lack of fluid inclusions in vein and vesicle minerals from the Izu–Bonin forearc/rear arc, and frequent occurrence and high diversity of non-carbonate vein minerals in the Troodos ophiolite.

Major vein calcite formation in the Troodos ophiolite probably occurred within an interval of ~ 10–20 Myr after crust formation. This coincides with major normal and detachment faulting in the Troodos SSZ that initiated contemporaneously with or shortly after Late Cretaceous spreading and channelized hydrothermal fluids (Varga and Moores 1985; Varga 1991; Bettison-Varga et al. 1992; Hurst et al. 1994; Varga et al. 1999). In contrast, reliable vein calcite ages in the Izu–Bonin forearc indicate vein formation between ~ 35 and ~ 33 Ma. This time interval coincides with normal faulting and assumed slab rollback in the Izu–Bonin forearc > 15 Myr after Early Eocene subduction initiation ~ 52 Ma following a hiatus (Christeson et al. 2016; Ishizuka et al. 2018; Robertson et al. 2018; Kurz et al. 2019; Reagan et al. 2019). Based on this temporal coincidence of calcite formation and normal faulting in both study areas, we hypothesize that the tectonic regime exerts an influence on the time of major calcite vein formation in oceanic crust. Since some samples of the sample suite could not be dated reliably using ^87^Sr/^86^Sr stratigraphy due to fluid–rock interaction and double intersections, it can be speculated that vein calcite formation ages exist that contrast this model. However, vein calcites that could not be dated reliably tend to be younger than their ambiguous intersection ages suggest. This is due to the involvement of mantle-derived Sr reducing the ^87^Sr/^86^Sr sample ratio and consistently increasing ^87^Sr/^86^Sr seawater ratios since ~ 40 Ma. This supports relatively late formation of vein calcites in the Izu–Bonin forearc coinciding with normal faulting > 15 Myr after volcanic activity. Earlier secondary mineralization might be restricted to pervasive alteration of volcanic rock matrices as particularly observed among boninites from Site U1439 (Reagan et al. 2015). Moreover, clear and consistent microtextural evidence for a non-tectonic origin of veining lacks. Crosscutting vein relationships and the lack of cooling fracture-specific structures argue against significant thermal contraction, although it might be an important process in the uppermost pillow lavas. Mineralized jigsaw breccia patterns of blocky calcite veins from the Izu–Bonin forearc point to hydro-fracturing (Phillips 1972; Agar 1994; Harper and Tartarotti 1996; Jébrak 1997; Tartarotti and Pasquaré 2003). Hydro-fracturing requires a low-permeable environment in which the fluid pressure exceeds the confining pressure (Jébrak 1997). Low-permeable sediments or sheet flows overlying the pillow lavas (Tartarotti and Pasquaré 2003) or pervasively altered rock matrices (Fisher 1998) represent such an environment. Regarding veins with well-developed jigsaw puzzle patterns from the Izu–Bonin forearc, ^87^Sr/^86^Sr stratigraphy suggests that brecciation and cementation occurred after a hiatus of > 15 Myr. This probably provided enough time to alter the host rock pervasively reducing its permeability.

Despite the different temporal framework of major calcite veining, reliable calcite vein formation ages in both study areas consistently fall within intervals of < 25 Myr after respective crust formation. This agrees with previous studies on alteration of oceanic crust in general (e.g., Hart and Staudigel 1978; Richardson et al. 1980; Staudigel et al. 1981; Staudigel and Hart 1985; Hart et al. 1994; Coogan and Gillis 2018).

The respective temporal framework of calcite veining proposed in this study also explains the differences in peak precipitation temperatures and mineralogy between mineralized veins from the Troodos ophiolite and the Izu–Bonin forearc/rear arc. Early tectonic extension shortly after emplacement of the Troodos pillow lavas facilitated deep fluid flow along faults and fluid heating in depth; whereas, late fracturing within the cold Izu–Bonin forearc crust did not result in significant heating of downflowing fluids. Moreover, structural analyses of ore deposits and hydrothermal sediments in the Troodos ophiolite indicate that hydrothermal circulation occurred in off-axis settings of the Troodos SSZ crust (e.g., Schiffman et al. 1987; Schiffman and Smith 1988; Eddy et al. 1998; Prichard and Maliotis 1998) extending the reach of crust modified by hydrothermal circulation. Therefore, localized high-T (> 100 °C) conditions indicated by high-T fluid inclusions entrapped in quartz, calcite, and analcime, the frequent non-carbonate mineral assemblage (quartz, analcime, heulandite, mordenite) indicative of elevated to high temperatures (~ 75–300 °C; Chipera and Apps 2001), and positive Eu anomalies of high-T blocky calcites are restricted to the Troodos ophiolite. In contrast, peak precipitation temperatures of vein calcites in the Izu–Bonin forearc and rear arc did not exceed 100 °C. This is in accordance with the absence of two-phase (liquid and vapor) fluid inclusions and the occurrence of phillipsite and/or harmotome, argillaceous vein selvedges, and palagonite. In general, fluid inclusions typically do not nucleate vapor bubbles at temperatures < 50 °C (Pagel et al. 2018) and phillipsite, harmotome, argillaceous minerals, and palagonite form at temperatures < 75 °C (Inoue 1995; Chipera and Apps 2001; Stroncik and Schmincke 2002). As similarly proposed by Alt et al. (2018), deep fluid exchange along extensional faults also explains the negative δ^13^C trend observed among vein calcites from the Troodos ophiolite. Faults probably tapped the δ^13^C mantle reservoir that is characterized by a bimodal δ^13^C distribution with peaks at − 5 and − 25‰ VPDB (Deines 2002). This might have induced mixing with seawater resulting in the observed δ^13^C values up to − 13.5‰ VPDB (Fig. 5). Similar δ^13^C values (− 9.7 to − 15.7‰ VPDB) were observed in secondary calcites hosted in sheeted dykes and gabbroic cumulates from the Troodos ophiolite (Vibetti 1993).

Admittedly, the comparability of outcrop (Troodos ophiolite) and drill core data (Izu–Bonin forearc/rear arc) is sophisticated since both approaches cover different dimensions (i.e., horizontal vs. vertical). The comparison of Sites U1439–U1441 with Site 786 that are separated by ~ 500 km allow a test of horizontal continuity, which shows that both localities are characterized by similar mineralogical and stable isotopic features. Moreover, resembling geochemical distribution patterns and the variety of vein microtextures equally observed among veins from outcrops and drill cores suggest representative drill core data. We are, therefore, confident that our comparative approach is applicable.

## Conclusions

Based on the comparison of published petrographic, mineralogical, and geochemical data of pillow lava-hosted mineralized veins from the Troodos and Izu–Bonin SSZ crust, this study yields the following findings.

- All study areas are predominantly pervaded by veins associated with extension fracture-controlled advective fluid flow. Depending on the mineral growth dynamics, these veins developed primarily blocky (precipitation into fluid-filled fractures) and secondarily syntaxial (crack and sealing) microtextures.
- Fluid diffusion-fed and crystallization pressure-driven antitaxial fiber veins occur in all study areas. They are particularly abundant relative to blocky veins in low-permeable crust and seem to become more important with crustal aging.
- Independently of the vein type, calcite is the major vein mineral. Non-carbonate minerals such as quartz and zeolites are particularly abundant in blocky and syntaxial veins from the Troodos ophiolite.
- In response to the different vein growth dynamics, calcites developed vein type-specific REE + Y characteristics. Increased fluid residence times and diffusive fluid supply intensified fluid-rock interaction. In contrast, crack and sealing prevented extensive fluid–rock interaction and resulted in pristine seawater signatures. Independent of vein type and study area, most secondary calcites precipitated from seawater to seawater-like fluids at temperatures < 50 °C emphasizing the importance of low-T seawater-mediated crustal alteration.
- Reliable ages of vein calcite precipitation in the respective areas coincide with Late Cretaceous (Troodos SSZ) and Early Eocene (Izu–Bonin forearc) extensional tectonic phases indicating a tectonic control on the time of calcite vein formation. In addition, microtextural analysis of calcite veins associated with rock brecciation indicate that fracturing was partly assisted by hydro-fracturing.
- Early tectonic extension shortly after emplacement of the Troodos pillow lavas and off-axis hydrothermal activity facilitated deep fluid flow along faults and fluid heating at depth. Late fracturing within the cold Izu–Bonin forearc crust, however, did not result in significant heating of downflowing fluids. Therefore, high peak precipitation temperatures (up to ~ 230 °C), high-T (up to ~ 300 °C) non-carbonate mineral assemblages, and mantle-derived δ^13^C values are restricted to the Troodos ophiolite.
- Higher peak fluid temperatures favored the entrapment of two-phase fluid inclusions in calcite, quartz, and analcime, and resulted in positive Eu anomalies of high-T blocky calcites. These well-preserved high-T features lack in the Izu–Bonin forearc/rear arc and suggest that uplift and emplacement of the Troodos ophiolite did not significantly overprint or alter the geochemical compositions of vein calcites.

## Data Availability

Data are archived at PANGAEA Data Archiving and Publication (Quandt et al. 2020b, https://doi.pangaea.de/10.1594/PANGAEA.920681).
